# Impact of age, heart rate, and body surface area on myocardial native T1, T2, and extracellular volume fraction in pediatric patients at 1.5 Tesla cardiac magnetic resonance imaging

**DOI:** 10.1007/s00247-026-06714-y

**Published:** 2026-07-30

**Authors:** Lucia Riaza-Martin, Jose-Miguel Escudero-Fernandez, Luis Riera-Soler, Roger Esmel-Vilomara, Hug Cuellar-Calabria, Elida-Josefa Vazquez-Mendez, Ferran Roses-Noguer, Anna Sabate-Rotes

**Affiliations:** 1 Department of Paediatrics, Obstetrics and Gynaecology, and Preventive Medicine and Public Health., Universitat Autònoma de Barcelona, Passeig de la Vall d’Hebron 119–129, Barcelona, Spain; 2 Department of Diagnostic Imaging and Nuclear Medicine, Vall d’Hebron University Hospital, Vall d’Hebron Barcelona Hospital Campus, Passeig de la Vall d’Hebron 119–129, 08035 Barcelona, Spain; 3 Institut de Diagnòstic per la Imatge (IDI), Barcelona, Spain; 4 Molecular Imaging and Therapy Research Group, Vall d’Hebron Research Institute (VHIR), Vall d’Hebron University Hospital, Vall d’Hebron Barcelona Hospital Campus, Passeig de la Vall d’Hebron 119–129, 08035 Barcelona, Spain; 5 Department of Pediatric Cardiology, Vall d’Hebron University Hospital, Vall d’Hebron Barcelona Hospital Campus, Passeig de la Vall d’Hebron 119–129, 08035 Barcelona, Spain; 6 Department of Pediatric Cardiology, Hospital de la Santa Creu i Sant Pau, Sant Pau Research Insitute (IR Sant Pau), Barcelona, Spain; 7 Department of Medicine, Universitat Autònoma de Barcelona, Barcelona, Spain

**Keywords:** Child, Heart rate, Magnetic resonance imaging, Myocardium, Pediatric cardiology, Reference values, Tissue characterization, Extracellular space

## Abstract

**Background:**

Cardiac magnetic resonance (CMR) enables the quantitative characterization of myocardial tissue through parametric myocardial tissue mapping. It shows a strong correlation with invasive histologic techniques in assessing diffuse myocardial fibrosis using native T1 mapping and extracellular volume (ECV), as well as in assessing myocardial edema with T2 mapping. Pediatric applicability is currently limited by a lack of specific reference values for this population, with no recommendations available from the Society of Cardiac Magnetic Resonance.

**Objective:**

To determine reference values for global left ventricular native T1, T2 mapping, and extracellular volume fraction and to assess the influence of age, heart rate, body surface area, and sex on these parameters in children and adolescents.

**Materials and methods:**

Data were retrospectively collected between 2018 and 2024. A total of 194 healthy children aged up to 17 years underwent CMR. The participants had a mean age of 12.6 (3.4) CI [12.1; 13.1] years (range 0.3–17), and 41% were female. A 1.5-T Siemens MAGNETOM Avanto Fit scanner was used. Native T1 mapping was performed at the base, mid-chamber, and apex of the left ventricle in the short-axis view at end-diastole using a MOLLI sequence, before and 15 min after the administration of a gadobutrol-based contrast agent. T2 mapping was performed before contrast administration at the base, mid-chamber, and apex of the left ventricle at end-diastole using a dark blood turbo spin echo sequence featuring a T2 preparation pulse and a balanced steady-state free precession (bSSFP). Linear regression models were employed to explore the relationships between native T1, ECV, and T2 mapping values and heart rate, age, and body surface area (BSA).

**Results:**

The results showed a mean global native T1 of 1004±37 ms, ECV of 25.9±3.6%, and T2 of 48.8±4 ms. T1 and ECV values were negatively correlated with age and BSA (*P*<0.001) and positively correlated with heart rate (*P*<0.001). In contrast, T2 values were negatively correlated with age (*P*<0.001) but not with heart rate (*P*=0.953 in the septum and *P*=0.445 in the free wall). Native T1 values were significantly higher in female patients than in males when analyzed separately for the interventricular septum and the left ventricular free wall (*P*=0.014 and *P*=0.001).

**Conclusion:**

This study provides a median range of values in native T1, T2 mapping, and ECV in a pediatric population. The low number of participants under the age of 6 should be taken into account in the results of our study. Our findings corroborate that native T1 is influenced by both age and heart rate, while T2 is primarily affected by age alone. We observed higher native T1 values in females, highlighting the need for separate, sex-specific reference ranges. Therefore, age, heart rate, and sex should be considered in pediatric myocardial mapping.

**Supplementary Information:**

The online version contains supplementary material available at https://doi.org/10.1007/s00247-026-06714-y.

## Introduction

Cardiac magnetic resonance (CMR) imaging enables the quantitative characterization of myocardial tissue through parametric tissue mapping [1]. Native T1 relaxation time and extracellular volume fraction (ECV) are used as non-invasive biomarkers for diffuse myocardial fibrosis, and show strong correlation with invasive histological techniques [2]. Myocardial T2 relaxation time can be used as a non-invasive biomarker to assess myocardial edema [2, 3].

These techniques are widespread in adult cardiology [4]. The 2018 updated Lake Louis criteria for diagnosing myocarditis in adults include an increase in T1 relaxation time as an indicator of myocardial injury and an increase in T2 relaxation time as an indicator of myocardial edema [5]. Tissue mapping is increasingly used for the same indications in the pediatric population [6], as well as for congenital heart disease [3, 6, 7].

The clinical and scientific applicability of these measures in children is limited by the absence of established reference values. The Society for Cardiovascular Magnetic Resonance (SCMR) released a scientific statement endorsed by the European Association for Cardiovascular Imaging (EACVI) [8] on how to conduct and interpret tissue mapping imaging in the pediatric population; they recommended using normal values tailored to the specific magnetic resonance imaging (MRI) scanner and sequence employed at each institution. However, due to the limited number of CMR studies performed on healthy pediatric patients, developing these reference values has been a challenge [9].

In recent years, numerous articles have been published focusing on reference values in adults [10, 11]. Fewer studies have been published on reference values in the pediatric population, and none has addressed the importance of age or heart rate when establishing specific thresholds for the aforementioned parameters [9, 12].

This study aims to establish pediatric global left ventricular (LV) reference values for native T1 and T2 mapping and ECV. Additionally, we aim to analyze the linear correlation of these parametric myocardial tissue mapping values with age, gender, body surface area (BSA), and heart rate. It should be emphasized that the values reported in this study may not be generalizable to other providers or to different techniques.

## Materials and methods

### Study population

**Fig. 1:**
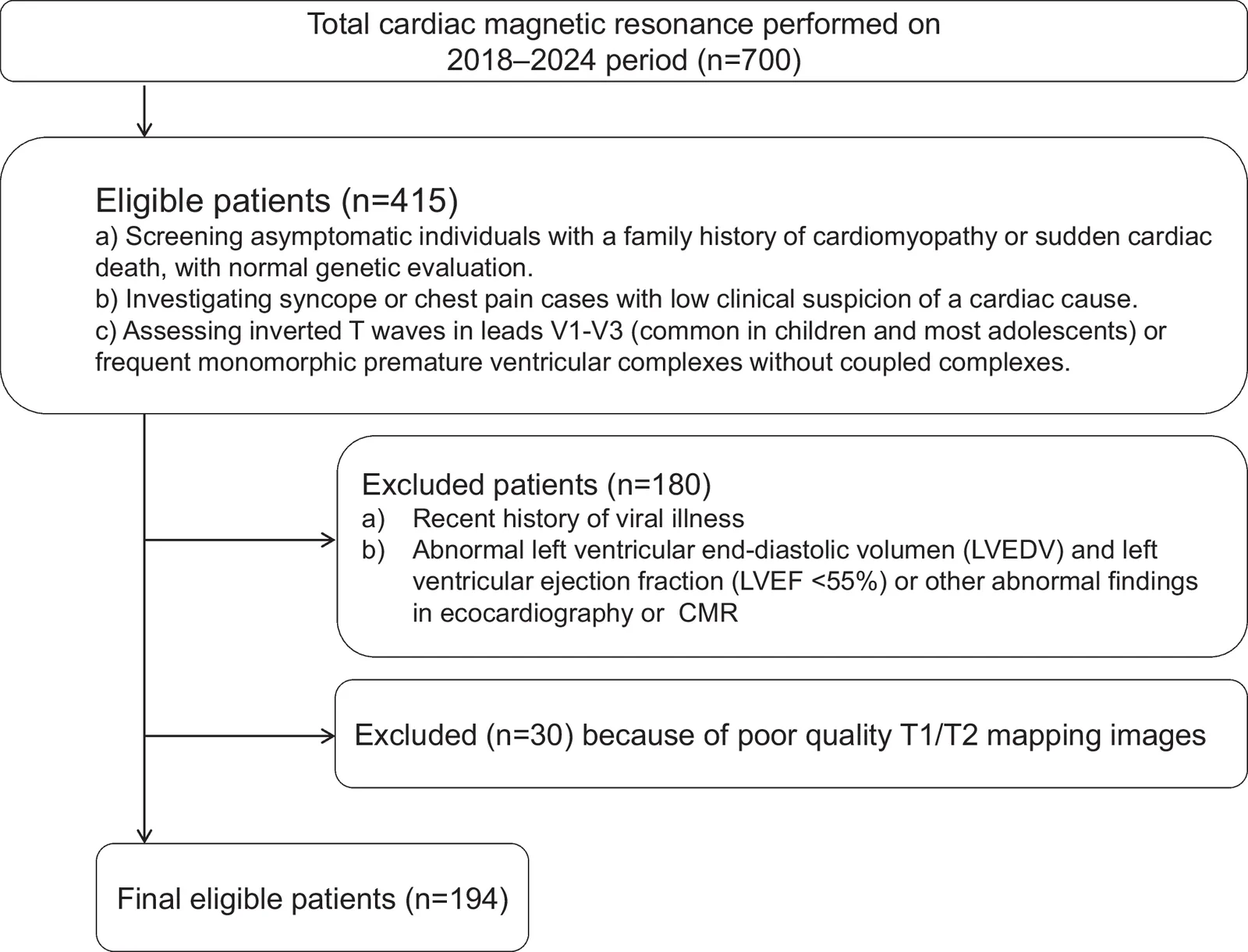
Flowchart for patient selection with inclusion and exclusion criteria

Data were retrospectively acquired from 2018 to 2024 and analyzed. A total of 415 healthy pediatric patients aged 0–17 years underwent CMR at a tertiary university hospital (Hospital Universitari Vall d’Hebron, Barcelona, Spain). Of those, 194 individuals were selected for analysis. A flowchart of the selection process with inclusion and exclusion criteria is presented in Fig. 1.

The most common indication for conducting CMR in healthy children was the screening of asymptomatic individuals with a positive family history. Other reasons included the investigation of abnormal T waves or premature ventricular contractions and syncope with low clinical suspicion of a cardiac cause.

Patients were categorized into seven groups according to age 0–3 years (*n*=2), 3–6 years (*n*=2), 6–9 years (*n*=22), 9–11 years (*n*=29), 11–13 years (*n*=32), 13–15 years (*n*=35), and 15–17 years (*n*=72). They were also grouped into three categories according to heart rate: 30–60 bpm (*n*=50), 61–90 bpm (*n*=118), 91–120 bpm (*n*=25). Comparative analysis between genders was performed on the entire cohort, rather than by categorized age groups, as there were not enough subjects in each group.

This study was approved by the research ethics board committee at Hospital Universitari Vall d´Hebron Hospital (study number 1000053256). The need for informed consent was waived.

### Cardiac magnetic resonance

CMR was conducted using a 1.5-T MRI scanner (MAGNETOM Avanto Fit scanner, software release VB17 and VE11B, Siemens Healthinners, Erlangen, Germany). A phased-array flexible surface coil was used for signal reception and a system body coil for radiofrequency transmission.

Ventricular volumes, function, and myocardial mass were assessed using balanced steady-state free precession (bSSFP) imaging, acquired as a short-axis stack covering the entire heart. The imaging parameters were set as follows: minimal repetition (TR) and echo times (TE); a flip angle of 70°; in-plane spatial resolution ranging between 1.5 mm^2^ and 2.0 mm^2^; slice thickness of 5 mm; the interslice gap adjusted to ensure coverage of both ventricles, typically requiring at least nine slices; and temporal resolution to reconstruct 20 true frames per cardiac cycle.

Native T1 relaxation time was measured at the midventricular level in the short-axis plane during diastole using a MOLLI sequence with a 5(nHB)3 configuration, meaning the number of heartbeats (nHB) varied between 3 and 5 depending on the heart rate. This ensured adequate T1 recovery between the two inversion experiments. Inline motion correction was applied. The imaging parameters were as follows: slice thickness of 8 mm; flip angle of 35°; TE between 1.13 ms and 1.26 ms; TR ranging from 2.68 ms to 2.95 ms; bandwidth of 1085 Hz/pixel; and a minimum inversion time (TI) between 100 ms and 120 ms, with 80-ms increments. Generalized autocalibrating partially parallel acquisitions (GRAPPA) were employed with an acceleration factor of 2. For post-contrast T1 quantification, measurements were repeated 15 min after the administration of a gadolinium-based contrast agent, gadobutrol (Gadovist; Bayer Healthcare, Berlin, Germany). Gadobutrol was administered at a dose of 0.2 mmol per kilogram of body weight for patients weighing less than 30 kg and 0.15 mmol per kilogram for patients weighing 30 kg or more.

Post-contrast T1 relaxation time was measured at the midventricular level in the short-axis plane during diastole using a MOLLI sequence with a 4(1)3(1)2 configuration and inline motion correction.

T2 relaxation time was measured in the short-axis plane at three levels: base, mid-chamber, and apex. These images were acquired at end-diastole during free breathing, with incorporated motion correction to minimize artifacts. A T2-weighted dark blood turbo spin echo (TSE) sequence was used, combined with a T2 preparation pulse and bSSFP readout [13]. The T2 mapping utilized TEs of 0 ms, 24 ms, and 55 ms, standardized across all heart rates. Motion correction techniques were applied during image acquisition. Importantly, T2 maps were generated prior to the administration of contrast agents [14].

All patients underwent contrast administration, as the examinations were to rule out early signs of arrhythmogenic cardiomyopathy.

Breath-holds were employed in awake and cooperative patients older than 6 years old; free breathing or ventilation techniques were utilized for all other patients, including those under sedation. All patients younger than 6 years were sedated (*n*=4).

### Image analysis

**Fig. 2:**
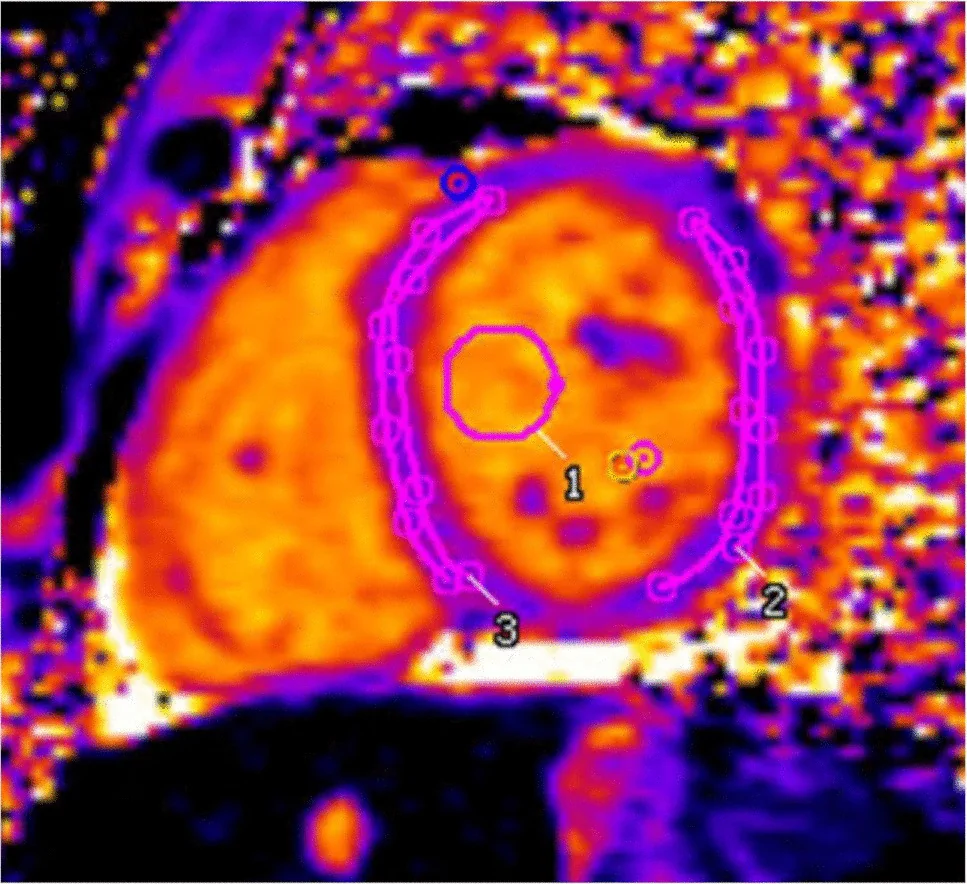
Representation of the ROI placement in short-axis mid-ventricular slice for determining myocardial native T1 value

Analysis of ventricular volumes, function, T1 and T2 relaxation times, and ECV was performed using a curve-fitting algorithm in commercially available software (CVI42, Circle Cardiovascular Imaging, Calgary, Canada). For T1 and T2 mapping, an average was derived from manually delineated regions of interest (ROIs) across three short-axis slices. A pediatric radiologist with 13 years of experience in CMR (L.R.M.) manually defined the ROIs. Representative ROIs were selected for the anteroseptal and inferoseptal segments of the interventricular septum (IVS; segments 8 and 9 of the American Heart Association’s 17-segment LV model) [15] and for the LV free wall, which includes anterolateral and inferolateral segments. To minimize partial volume effects at the myocardial borders, ROIs were confined to the wall, avoiding endocardial and epicardial borders. For blood T1 relaxation times—both pre- and post-contrast—ROIs were carefully delineated at the center of the LV cavity to exclude trabeculations and papillary muscles from the analysis (offset of 10%). The same method was applied for T2 relaxation time calculations (Figure 2).

ECV fraction for each ROI was calculated using pre- and post-contrast T1 relaxation times and the hematocrit obtained on the same day [16].

All analyses were conducted in accordance with the guidelines set by the SCMR for reporting CMR examinations. Cine short-axis image stacks were analyzed to obtain precise measurements of ventricular volumes with papillary muscles included [17]. All volumes and masses were indexed to the individual’s BSA, with z-scores derived based on reference data from Buechel et al. [18].

Volumetric analysis was conducted by three experienced observers (L.R.M., L.R.S., and A.S.R.), each with more than 10 years of experience in CMR. T1 and T2 quantification was performed by a single experienced observer (L.R.M.).

### Statistical analysis

Data were expressed as mean ± standard deviation or as counts with percentages, as appropriate. Paired Student’s *t*-tests were used to compare data within the same patient group. Between-group comparisons were performed with unpaired Student’s *t*-tests, Mann–Whitney *U* tests, ANOVA, or Kruskal–Wallis tests with Bonferroni correction, depending on whether the data followed a normal distribution.

Univariable linear regression was used to assess associations between myocardial native T1 relaxation time and ECV with other clinical parameters.

To assess reliability, intraclass correlation coefficients (ICC) were calculated for both interobserver and intraobserver comparisons. Intraobserver agreement was evaluated by having the first author (L.R.M.) reanalyze 10 datasets 1 month later, with all contours redrawn. To assess interobserver reliability, a second independent observer (A.S.R.) repeated the analysis while blinded to the initial results. Further reliability analysis was conducted using Bland–Altman plots with limits of agreement.

Statistical significance was determined at a threshold of *P*<0.05. Statistical analysis was carried out in the Statistics and Bioinformatics Unit (UEB) at Vall d’Hebron Hospital Research Institute (VHIR) using R statistical software (version 4.3.0; The R Foundation for Statistical Computing, 2015).

## Results

A total of 194 patients met the inclusion criteria and were included in the study, with a median age of 11.26 (IQR 10–16) years; 58.8% were male. Baseline characteristics of the study group and results from volumetric analysis are presented in Table 1. All participants had normal LV size, mass, and systolic function. The median heart rate was 70.6 (±16.3) beats per minute (range 59–81).

**Table 1:** Demographic, mass, and volumes for all healthy subjects and according to gender

	**All (*n*=205)**	**Male (*n*=118)**	**Female (*n*=87)**	*P*-value*
Age (y)	12.9±3.5	12.3±3.6	12.1±3.7	0.657
Height (cm)	156.4±20.6	159.3±21.9	150.1±17.3	0.056
Weight (kg)	51±18.4	51.3±18.7	43.1±16.5	0.055
BSA (m^2^)	1.5±0.4	1.5±0.4	1.3±0.3	0.449
Heart rate (bpm)	70.3±16.3	64.3±14.8	73.8±16.4	0.065
LV mass (g/m^2^)	56.1±14.3	60.3±15.3	48.3±10	0.024
LVEDVi (mL/m^2^)	82.7±16.4	87.5±15.5	73.8±13.8	0.087
LVESVi (mL/m^2^)	30.9±9.2	32.1±9.1	27.1±8.5	0.124
LVEF (%)	62.5±8.2	61.7±7.9	61.5±8.5	0.658
LV mass/volume (g/mL)	0.67±0.15	0.69±0.16	0.65±0.14	0.242
RVEDVi (mL/m^2^)	77.1±19.2	84.3±18.5	67.3±15.4	0.040
RVESVi (mL/m^2^)	35±12.1	30.1±10	38.6±12	0.034
RVEF (%)	55±9.8	55.4±10.6	54.4±9.2	0.036

*1*, ROI located within the cavity; *2*, ROI located on the free wall; *3*, ROI located on the interventricular septumsurface area; *LV*, left ventricle; *LVEDVi*, indexed left ventricular end-diastolic volume; *LVESVi*, indexed left ventricular end-systolic volume; *LVSVi*, indexed left ventricular stroke volume; *LVEF*, left ventricular ejection fraction; *RVEDVi*, indexed right ventricular end-diastolic volume; *RVESVi*, indexed right ventricular end-systolic volume; *RVSVi*, indexed right ventricular stroke volume; *RVEF*, right ventricular ejection fraction

*P-values represent comparisons between male and female subjects

CMR results, including T1 and T2 mapping and ECV, are presented according to sex in Table 2. The mean global native T1 relaxation time was 1004±37 ms, mean global ECV was 25.9±3.6%, and the mean T2 relaxation time was 48.8±4 ms.

**Table 2:** Native T1, ECV, and T2 values for all healthy subjects and according to gender

	**All (*n*=194)**	**Male (*n*=114)**	**Female (*n*=80)**	*P*-value*
Global native T1 (ms)	1,004.4±37	994.6±67.1	1,017.2±40.2	0.066
IVS native T1 (ms)	1,011.6±39.1	1,000.4±36.2	1.024.6±41.3	0.014
Free wall native T1 (ms)	997.2±40.5	969±86.4	1,009.9±37.9	0.001
Global ECV (%)	25.9±3.6	25±3.5	27.1±3.5	0.125
IVS ECV (%)	26.9±3.6	25.8±3.5	27.7±3.3	0.264
Free wall ECV (%)	25.7±3.7	24.3±3.3	26.5±3.7	0.174
Global T2 (ms)	48.8±4	47.8±4.1	49.5±4.3	0.478
IVS T2 (ms)	49.2±4.6	48±4.4	49.9±4.2	0.576
Free wall T2 (ms)	48.5±4.1	47.6±4.1	49.1±4.3	0.573
Blood T1 (ms)	1,589.7±70.3	1,571.2±75.9	1,606±57	0.013
Hematocrit (%)	40.4±3.8	41.4±4.2	39.2±2.8	0.034

*IVS*, interventricular septum

*P-values represent comparisons between male and female subjects

When categorized into age groups, ECV, native T1, and T2 relaxation times were higher in younger patients and decreased with age (Table 3). Specifically, the oldest group (15–17 years) had significantly lower T1 and T2 values compared to the youngest in both the IVS (*P*=0.029) and LV lateral wall (*P*<0.001).

**Table 3:** Distribution of native T1, ECV and T2 values at the interventricular septum and free wall of left ventricle, according to age group

	**0–3 (*n*=2)**	**3–6 (*n*=2)**	**6–9 (*n*=22)**	**9–11 (*n*=29)**	**11–13 (*n*=32)**	**13–15 (*n*=35)**	**15–17 (*n*=72)**	*P-*value*
Global native T1 (ms)	1,156±12	1,042.3±24.5	1,026.9±50.8	1,012.3±34.8	999.4±35.6	999.9±33.8	979.2±79.8	0.006
IVS native T1 (ms)	1,148.5±12	1,042.5±13.4	1,033.8±55.9	1,013±27.6	1,002.5±37.2	1,011.2±34.4	1,002.7±32.3	0.007
Free wall native T1 (ms)	1,163±7.8	1,042.1±40.3	1,020±45.4	1,011.5±41.3	996.2±34.3	988.6±29.5	956.2±106	0.004
Global ECV (%)	35.8±2.6	38.6±4.1	29.1±3.3	27.2±2.3	26.1±2.6	24.9±2.1	24.7±3.5	0.009
IVS ECV (%)	36.3±1.7	37.1±5.6	29.9±3	27.4±2.3	26.7±2.4	25.5±1.8	25.6±3.7	0.008
Free wall ECV (%)	35.2±4	40.1±3.2	28.4±3.6	27.1±2.2	25.6±2.7	24.3±2.2	23.8±2.9	0.006
Global T2 (ms)	60.5±2.8	58.7±3.8	49.8±4.7	49.6±4	48.8±3.7	47.6±3.6	47.6±3.8	0.040
IVS T2 (ms)	62.3±3.3	58.4±4.9	50.4±5.2	50.3±4	49.6±3.6	47.7±3.7	47.5±3.9	0.035
Free wall T2 (ms)	58.8±0.3	58.9±4.2	49.2±4.2	48.9±4	48±3.7	47.5±3.5	47.6±3.8	0.042

Age in years

*IVS*, interventricular septum

*P-values represent comparisons between eight subgroups using Kruskal–Wallis test with Bonferroni corrections

**Fig. 3:**
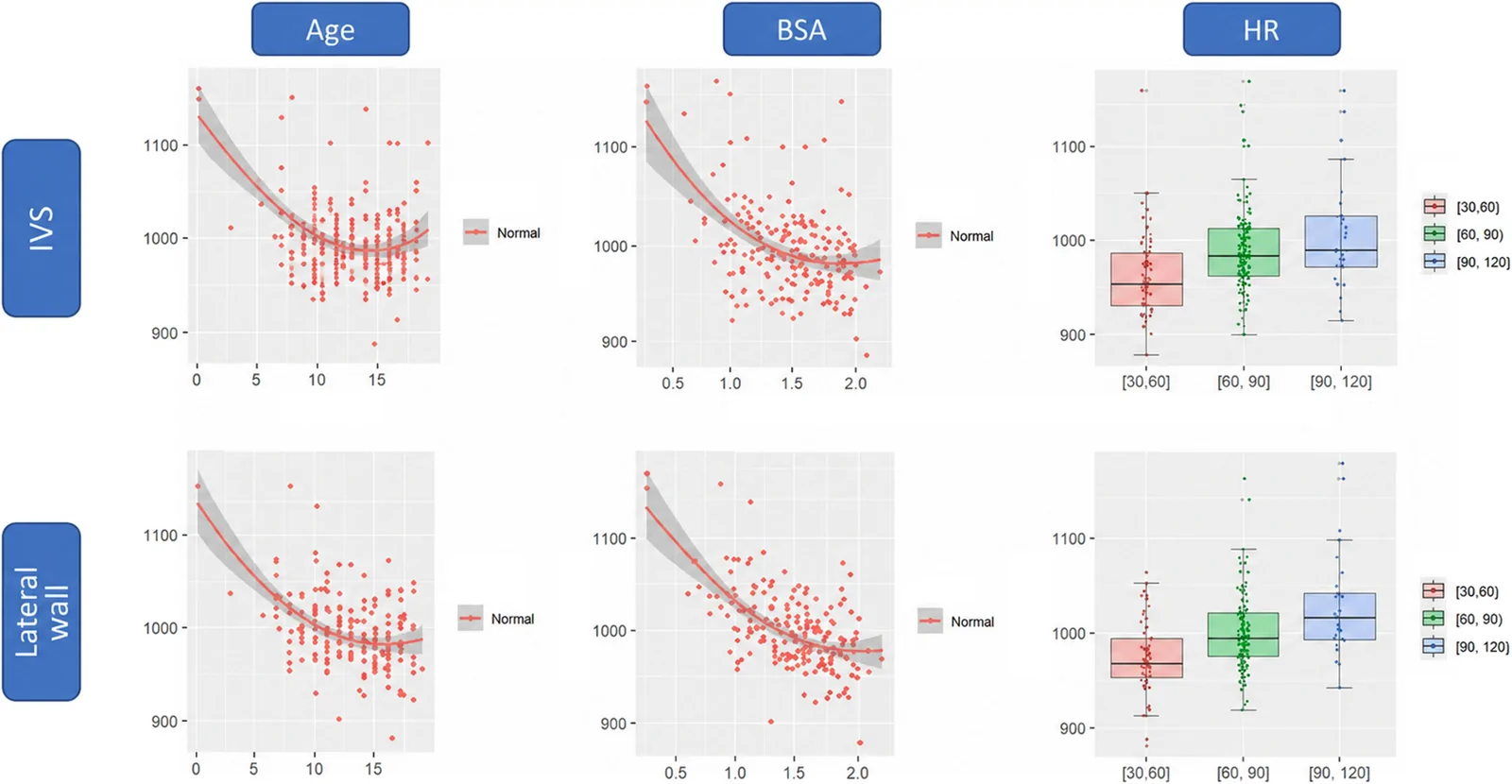
Univariate regression analysis models that represent distribution of native T1 values at the interventricular septum and free wall according to age, BSA, and HR (graphically)

**Fig. 4:**
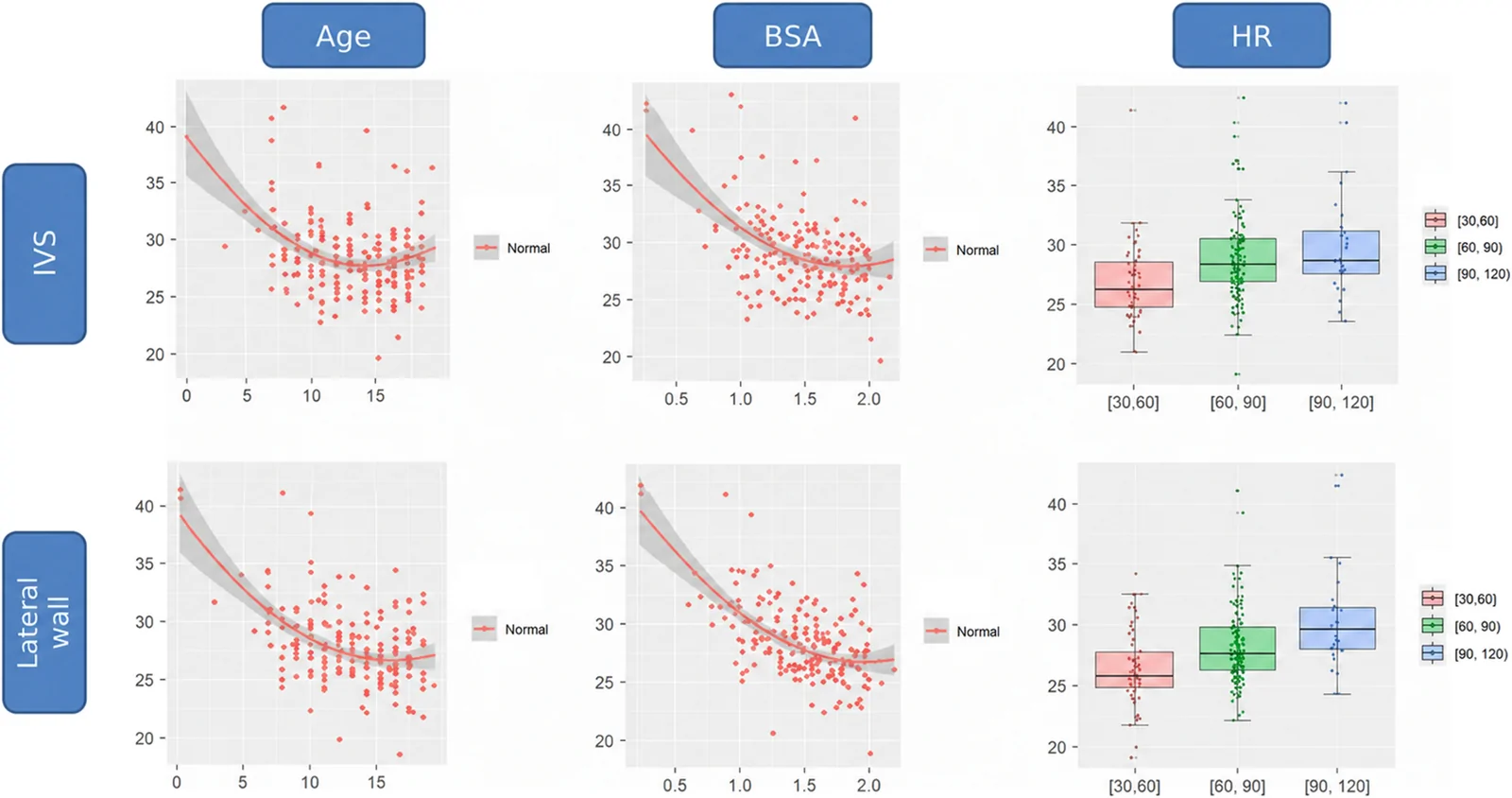
Univariate regression analysis models that represent distribution of ECV values at the interventricular septum and free wall according to age, BSA, and HR (graphically)

**Fig. 5:**
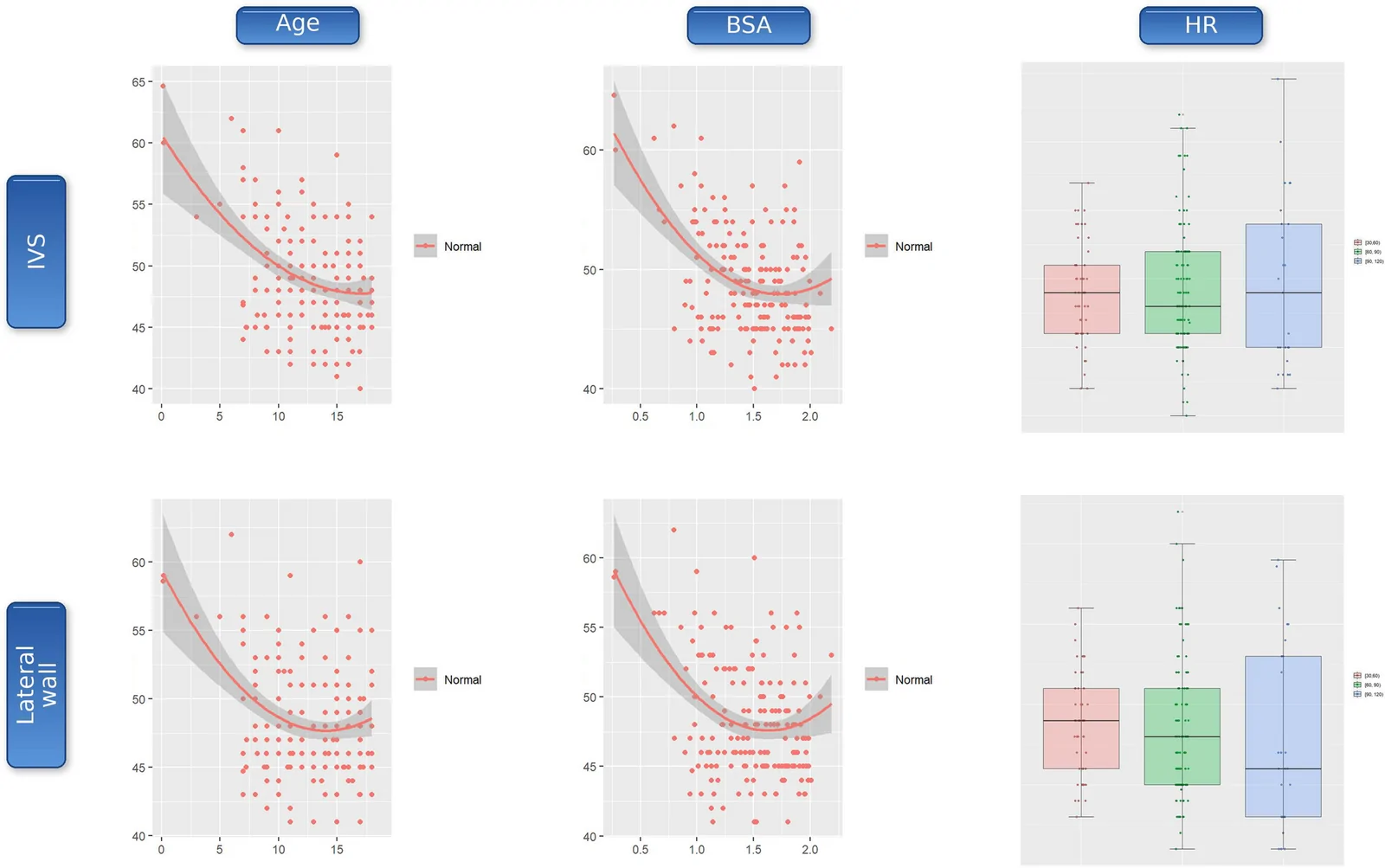
Univariate regression analysis models that represent distribution of T2 values at the interventricular septum and free wall according to age, BSA, and HR (graphically)

When subjects were categorized into three groups according to heart rate, T1 relaxation time and ECV were higher in those with the highest heart rates. No significant differences were observed in T2 values (Table 4 and Supplementary Table 1; Figs. 3, 4, and 5).

**Table 4:** Distribution of native T1, ECV, and T2 values at the interventricular septum and free wall of left ventricle, according to heart rate

	**0–3 (*n*=2)**	**3–6 (*n*=2)**	**6–9 (*n*=22)**	**9–11 (*n*=29)**	**11–13 (*n*=32)**	**13–15 (*n*=35)**	**15–17 (*n*=72)**	*P-*value*
Global native T1 (ms)	1,156±12	1,042.3±24.5	1,026.9±50.8	1,012.3±34.8	999.4±35.6	999.9±33.8	979.2±79.8	0.006
IVS native T1 (ms)	1,148.5±12	1,042.5±13.4	1,033.8±55.9	1,013±27.6	1,002.5±37.2	1,011.2±34.4	1,002.7±32.3	0.007
Free wall native T1 (ms)	1,163±7.8	1,042.1±40.3	1,020±45.4	1,011.5±41.3	996.2±34.3	988.6±29.5	956.2±106	0.004
Global ECV (%)	35.8±2.6	38.6±4.1	29.1±3.3	27.2±2.3	26.1±2.6	24.9±2.1	24.7±3.5	0.009
IVS ECV (%)	36.3±1.7	37.1±5.6	29.9±3	27.4±2.3	26.7±2.4	25.5±1.8	25.6±3.7	0.008
Free wall ECV (%)	35.2±4	40.1±3.2	28.4±3.6	27.1±2.2	25.6±2.7	24.3±2.2	23.8±2.9	0.006
Global T2 (ms)	60.5±2.8	58.7±3.8	49.8±4.7	49.6±4	48.8±3.7	47.6±3.6	47.6±3.8	0.040
IVS T2 (ms)	62.3±3.3	58.4±4.9	50.4±5.2	50.3±4	49.6±3.6	47.7±3.7	47.5±3.9	0.035
Free wall T2 (ms)	58.8±0.3	58.9±4.2	49.2±4.2	48.9±4	48±3.7	47.5±3.5	47.6±3.8	0.042

Heart rate in bpm (beats per minute)

*IVS*, interventricular septum

*P-values represent comparisons between three subgroups using ANOVA test with Bonferroni corrections

Significant sex differences were observed in native T1 values, with female patients exhibiting higher values than males in both the IVS (1024±41.3 ms vs 1000.4±36.2 ms; *P*=0.014) and lateral wall (1009.9±37.9 ms vs 969±86.4 ms; *P*=0.001).

The average native T1 relaxation time was not significantly higher in the IVS than in the lateral wall (1012 ms vs. 996.4 ms; *P*=0.141). The average ECV in the IVS (26.9%) was not significantly higher than in the free wall (25.6%, *P*=0.174). No significant changes were seen in either global or regional T2 values (*P*=0.124).

Global and regional T1, T2, and ECV values were inversely correlated with age and BSA. In contrast, heart rate was directly related to T1 and ECV, but did not show a relationship with T2 values.

Patients in younger age groups were associated with higher heart rates and lower BSA. The three traits were consistently related to values T1, T2, and ECV, except for heart rate and T2.

**Fig. 6:**
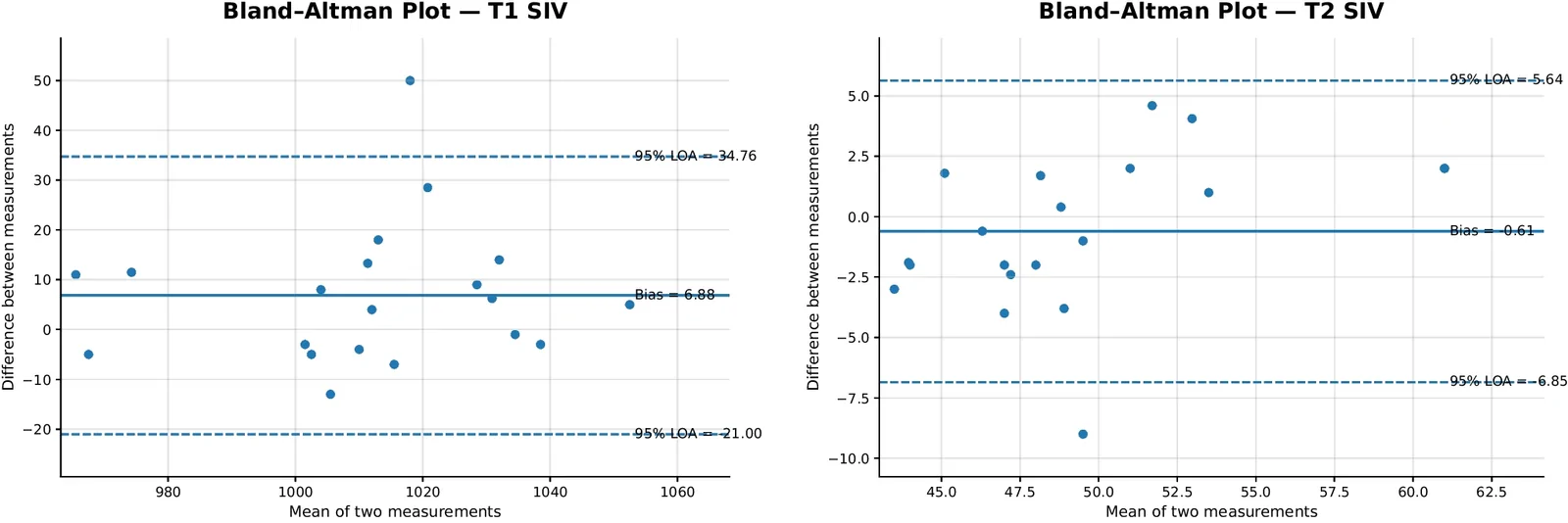
Intraobserver reliabilities for T1 and T2

**Fig. 7:**
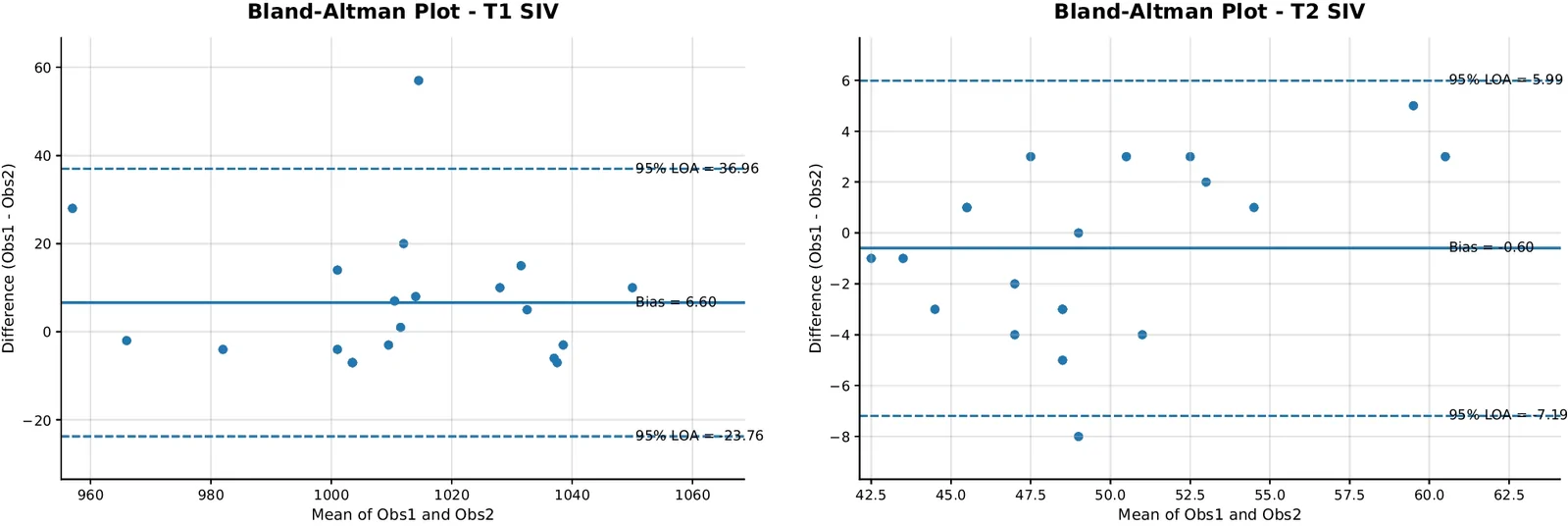
Interobserver reliabilities for T1 and T2

Inter- and intraobserver reliabilities for T1 and T2 values were excellent, with intraclass correlation coefficients (ICCs) consistently exceeding 0.75. Specifically, the interobserver ICC for T1 values was 0.81 (CI 0.58–0.92; *P*<0.001), and for T2 was 0.82 (CI 0.58–0.92; *P*<0.001). Intraobserver ICCs were 0.82 for T1 (CI 0.60–0.93; *P*<0.001) and 0.84 for T2 (CI 0.62–0.93; *P*<0.001). Further reliability analysis is presented in Bland–Altman plots in Figs. 6 and 7.

## Discussion

This investigation provides a range of pediatric values for myocardial native T1, ECV, and T2 mapping using 5(nHB)3 MOLLI acquisition and dark blood TSE sequences on a 1.5-T MRI scanner (Siemens MAGNETOM Avanto Fit Scanner), gathered in a single center.

To the best of our knowledge, this study is the first to describe native T1, T2, and ECV values together and their associations with age, BSA, and heart rate in a pediatric population. Previous pediatric articles addressing this topic in smaller groups have focused on either the relationship between T1 and T2 values, age, and heart rate [19], or on associations between T1 values, ECV, BSA, and heart rate [20].

The limited number of participants under 6 years of age should be considered when interpreting the results of this study.

While some studies have shown a correlation between age and native T1 mapping or ECV [21, 22], most reports describe no significant age-related variations in healthy adults [22–26] or children [9, 13]. However, we found a statistically significant, inversely proportional relationship with age for T1, T2, and ECV values.

It is unclear whether this difference is due to a larger extracellular matrix size in younger children or to methodological limitations relating to pulse sequences or ROI size.

The global ECV values observed in our study (25.9%) were consistent with those reported in adults using a 3(3)3(3)5 acquisition scheme, which ranged from 25% to 27% [24, 27].

Alsaied et al. [28] evaluated 113 healthy subjects with pectus excavatum, with an older age range of 5–19 years. A different MR scanner was used, but with the same MOLLI sequence and 5(nHB)3 configuration. Consistent with our results, they observed a weak negative correlation between native T1 values and age (*r*=−0.39, *P*<0.001) in univariable analysis. Age was independently associated with T1 in multivariable analysis. They also observed a weak negative correlation of T2 with age (*r*=−0.20, *P*=0.047).

Pagano et al. [29] evaluated 48 healthy subjects aged 9–18 years using a 1.5-T MRI scanner (Siemens MAGNETOM Avanto, software release VB17 and VE11B) with a 3(3)3(3)5 MOLLI sequence; the contrast material and acquisition time were the same as our study. They observed a correlation between ECV and age in univariate but not multivariate analysis.

The negative correlation between age and native T1 or ECV could be the result of changes in extracellular or cellular components.

The average T1 values in our youngest participants, who had faster heart rates, were higher than previously reported adult values using the MOLLI sequence, which have ranged from 950 ms to 982 ms [22, 24, 30]. This discrepancy may be attributed to our use of the 5(nHB)3 acquisition scheme in place of the “traditional” 3(3)3(3)5 configuration used in most adult studies. This reduces sensitivity to heart rate fluctuations, leading to higher T1 times compared to the original MOLLI scheme [26]. None of the previous articles using MOLLI sequencies reported pediatric ECV values. Meanwhile, heart rate was positively correlated with T1 and ECV but not with T2 values. The T2-mapping sequence does not have this dependency on heart rate because it is not based on inversion recovery, and so does not require these signal recovery periods.

Alsaied et al. [28] used a different 1.5-T MR scanner (Philips Ingenia) with the same MOLLI sequence and a 5(nHB)3 configuration. Consistent with our results, in their pediatric cohort, they observed a weak positive correlation with heart rate (*r*=0.32, *P*<0.001) in univariate analysis; this was independently associated with T1 in multivariable analysis. They similarly observed no correlation between T2 mapping and heart rate. Pagano et al. [29] observed a correlation between ECV and BSA in univariate analysis but not in multivariate analysis.

Reports on sex differences in myocardial native T1 values have been inconsistent across previous studies. Some describe higher values in adult females [22, 23, 25], while others report no significant differences [21, 22]. This variability extends to pediatric studies [9, 13]. In adults, sex-related differences in myocardial remodeling are well-documented [15], including variations in LV values [26] and histologic findings [31]. These differences may be influenced by hormonal factors [15]. The sex differences we observed in blood T1 values may be related to differences in hematocrit levels, which can impact myocardial T1. These findings suggest the need for distinct reference values for myocardial native T1 and ECV in male and female pediatric patients.

In our study, native myocardial T1 values were significantly longer in female patients compared to males when considering the septal (*P*=0.014) or free wall segments (*P*<0.001) separately, but not when considering the entire LV (*P*=0.066). Overall, the average native T1 in the IVS was higher than in the free wall (1012 ms vs 996.4 ms; *P*<0.001); however, this difference was found to only be significant in males (*P*<0.001). Additionally, average native T1 values were longer in females than males (*P*=0.013), likely due to lower average hematocrit levels in females (*P*=0.034). Alsaied et al. [28] found no differences between male and female pediatric patients in terms of T1 or T2 values. Similarly, Pagano et al. [28] reported no sex differences in native myocardial T1 and ECV.

A key study by Parekh et al*.* [24] involved a small control group of healthy children and young adults (*n*=21, predominantly male, age 12–18). They reported an average T1 relaxation time of 965.6 ms using similar imaging techniques to the present study. In line with our results, they observed a significant difference in T1 values between male and female patients. The slightly lower T1 values compared to those in our study may be attributed to the older age and higher proportion of male participants in the control group. More recently, the same group published on a larger healthy cohort with slightly higher values of native T1 (990 ms) and ECV (23.3%) [14].

In our study, we found no statistically significant differences in any of the three parameters analyzed (T1, T2, and ECV) when comparing the mean data between the interventricular septum and the lateral wall. Given its higher reproducibility and lower susceptibility to partial-volume effects and motion artifacts, septal quantification should remain the preferred approach for clinical myocardial mapping in children. Assessment of the lateral wall and whole-slice measurements may be useful for research purposes and for improving understanding of regional myocardial characteristics in the pediatric population.

We reported T1 and T2 values for the entire myocardium, and separate analysis of interventricular septum and lateral wall data, whereas most adult studies focus on the IVS; however, pediatric studies show no significant difference in measurements between the two [13].

Olivieri et al. [7] reported myocardial native T1 and ECV values in a small cohort of exclusively male patients with an average age of 16.1 years, using similar acquisition methods to our study. They provided native T1 septal and free wall values of 900±34 ms and 978±36 ms, respectively, but did not report whole LV values. Meanwhile, they observed higher septal and free wall ECV values (26.0%±3.3 and 24.4%±3.5, respectively).

Pagano et al. [29] observed increased myocardial native T1 and ECV values in the IVS vs the free wall, and Parekh et al*.* [24] observed lower T1 values in the lateral wall compared to the IVS.

Differences in methodologies likely contribute to these discrepancies. Overall, the finding that native T1 and ECV were elevated in the septum compared to the free wall aligns with previous studies [22, 28]. This finding is probably influenced by factors affecting the accuracy of T1 measurements in the free wall region, particularly field inhomogeneity at the air-tissue interface [32]. Therefore, current guidelines recommend using the IVS for T1 and ECV quantification, unless a specific pathology affects another region [8].

A comparison between the findings of this study and the existing literature is summarized in Table 5.

**Table 5:** Summary of the results of our study compared to bibliography

Study: parameters	Age	HR	BSA	Gender	IVS/lateral wall
Present study: T1, T2, and ECV values	Negative correlation	T1 and ECV: positive correlation T2: no relation	Negative correlation	Girls T1 longer	No differences
Alsaied et al: T1 and T2	Negative correlation	T1: positive correlation T2: no relation		No differences	
Pagano et al: T1 and ECV	Negative correlation		Negative correlation	No differences	Increased T1 and ECV in IVS
Oliveri et al:					Increased T1 and ECV in IVS
Parekh et al:				Girls T1 longer	Higher T1 in girls

## Limitations

The single-center, retrospective design may limit the applicability of these findings due to variability in equipment and sequence configurations between institutions [13].

Although our sample size is the largest reported in the literature on this topic [28, 29], the relatively small group sizes (fewer than 50) underscore the need for larger studies. These could establish more reliable reference values across different pediatric subpopulations. Younger ages are naturally less well represented, as MRI is less frequently conducted due to the need for anesthesia in most infants under 6 years of age. This may explain the low number of patients (*n*=2) in the 0–3-year and 3–6-year categories. The lack of younger participants also means a lower sample of children with higher heart rates, with only 12% of patients having a heart rate above 90 bpm in our cohort. The study also lacked data on the pubertal stage of participants; however, we stratified by age groups to mitigate any potential effects of this.

ROI size variability in smaller hearts in younger children may introduce partial volume effects and reduce measurement accuracy.

It should be noted that the 6–9-year-old age group is relatively heterogeneous in terms of breath-holding ability; therefore, the technique is tailored to individual characteristics to optimize image quality.

All subjects in this study were either asymptomatic, with a family history of cardiomyopathy or sudden cardiac death, or had electrocardiogram alterations or symptoms such as syncope or chest pain. However, despite their benign evolution after the negative CMR, cardiac involvement cannot be completely ruled out.

We also acknowledge that magnetization transfer effects might affect the MOLLI sequence, potentially impacting T1 accuracy [33].

## Conclusion

This study provides median range of values on native T1, T2 mapping, and ECV in a pediatric population. These can serve as reference values for institutions using the same MR scanner and the same MR pulse sequence technique. The small number of participants under 6 years of age should be considered when interpreting the study results.

Our findings corroborate that native T1 is influenced by both age and heart rate, while T2 is primarily affected by age alone. Consistent with studies in adult populations, we observed higher native T1 values in females, highlighting the need for separate, sex-specific reference ranges. Therefore, age, heart rate, and sex should be considered in pediatric myocardial mapping.

We observed no significant differences in native T1, T2, or ECV between the interventricular septum and the left ventricular lateral wall. Given its higher reproducibility and reduced susceptibility to artifacts, septal quantification should be considered the preferred reference site for clinical myocardial mapping in children. In this context, lateral wall and whole-slice measurements do not appear to add clinically relevant information, although they may still be useful for research applications and for the assessment of regional myocardial heterogeneity in specific disease settings.

## Supplementary Information

Below is the link to the electronic supplementary material.

ESM1 (DOCX 13.2 KB)

## Data Availability

No datasets were generated or analysed during the current study.
