# Correction: Impact of age, heart rate, and body surface area on myocardial native T1, T2, and extracellular volume fraction in pediatric patients at 1.5 Tesla cardiac magnetic resonance imaging

**DOI:** 10.1007/s00247-026-06813-w

**Published:** 2026-10-21

**Authors:** Lucia Riaza-Martin, Jose-Miguel Escudero-Fernandez, Luis Riera-Soler, Roger Esmel-Vilomara, Hug Cuellar-Calabria, Elida-Josefa Vazquez-Mendez, Ferran Roses-Noguer, Anna Sabate-Rotes

**Affiliations:** 1 Department of Paediatrics, Obstetrics and Gynaecology, and Preventive Medicine and Public Health, Universitat Autònoma de Barcelona, Passeig de la Vall d’Hebron 119–129, Barcelona, Spain; 2 Department of Diagnostic Imaging and Nuclear Medicine, Vall d’Hebron University Hospital, Vall d’Hebron Barcelona Hospital Campus, Passeig de la Vall d’Hebron 119–129, 08035 Barcelona, Spain; 3 Institut de Diagnòstic Per La Imatge (IDI), Barcelona, Spain; 4 Molecular Imaging and Therapy Research Group, Vall d’Hebron Research Institute (VHIR), Vall d’Hebron University Hospital, Vall d’Hebron Barcelona Hospital Campus, Passeig de la Vall d’Hebron 119–129, 08035 Barcelona, Spain; 5 Department of Pediatric Cardiology, Vall d’Hebron University Hospital, Vall d’Hebron Barcelona Hospital Campus, Passeig de la Vall d’Hebron 119–129, 08035 Barcelona, Spain; 6 Department of Pediatric Cardiology, Hospital de La Santa Creu I Sant Pau, Sant Pau Research Insitute (IR Sant Pau), Barcelona, Spain; 7 Department of Medicine, Universitat Autònoma de Barcelona, Barcelona, Spain

**Correction: Pediatric Radiology**

**https://doi.org/10.1007/s00247-026-06714-y**

The authors wish to report an error in the figures of the published article. During the production process, corrected versions of the figures were provided to the production team. However, the original versions of the figures were inadvertently published in the online version of the article. The figures should therefore be replaced with the corrected versions provided by the authors. The corrected figures do not affect the results, interpretation, conclusions, or scientific content of the article.

We apologize for any inconvenience caused by this error and thank the publisher for facilitating the correction.

Corrected figures:

Figures [3–5] should be replaced by the corrected versions provided with this Correction/Erratum.

**Figure:**
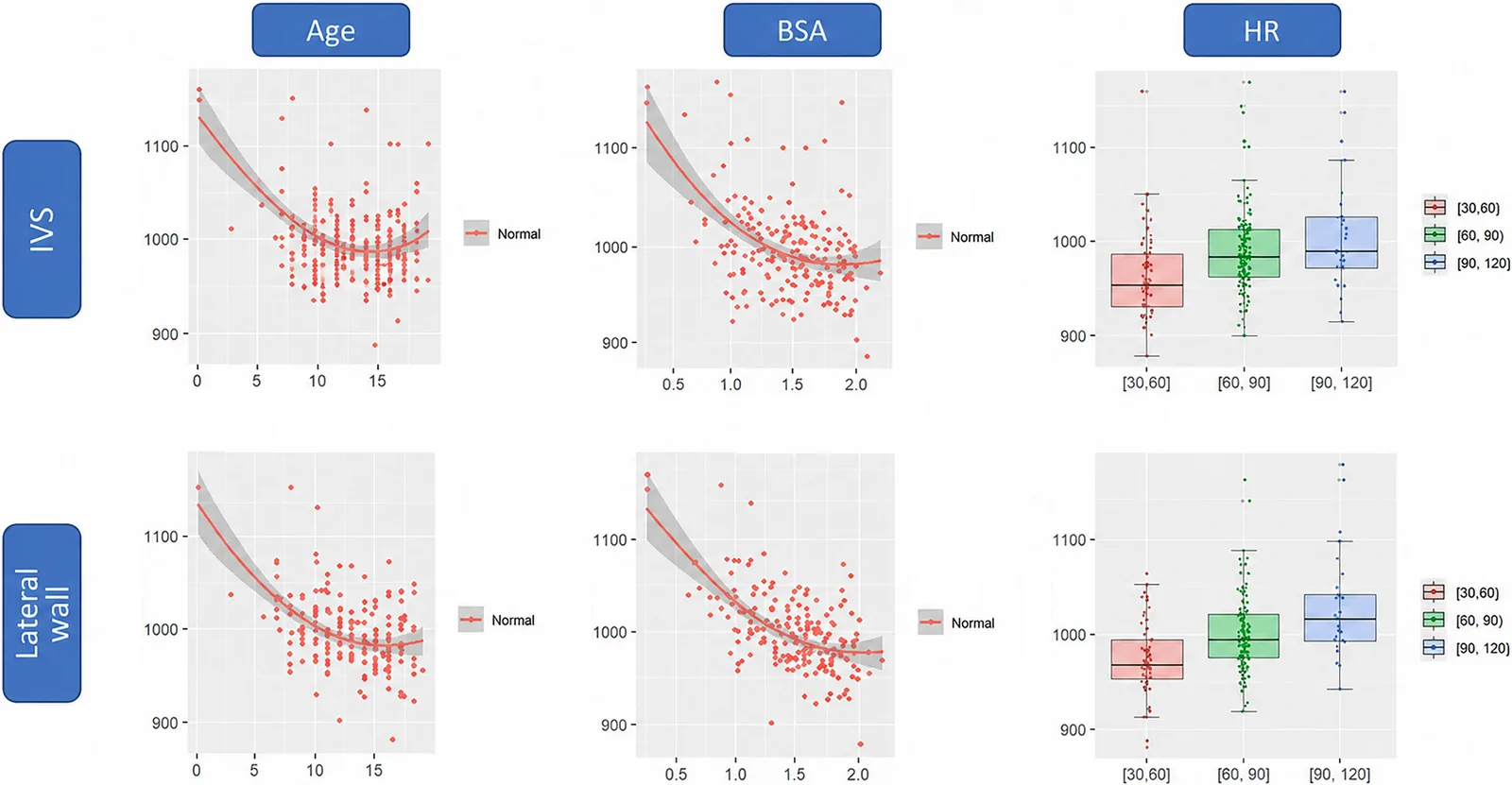

**Fig. 3**

**Figure:**
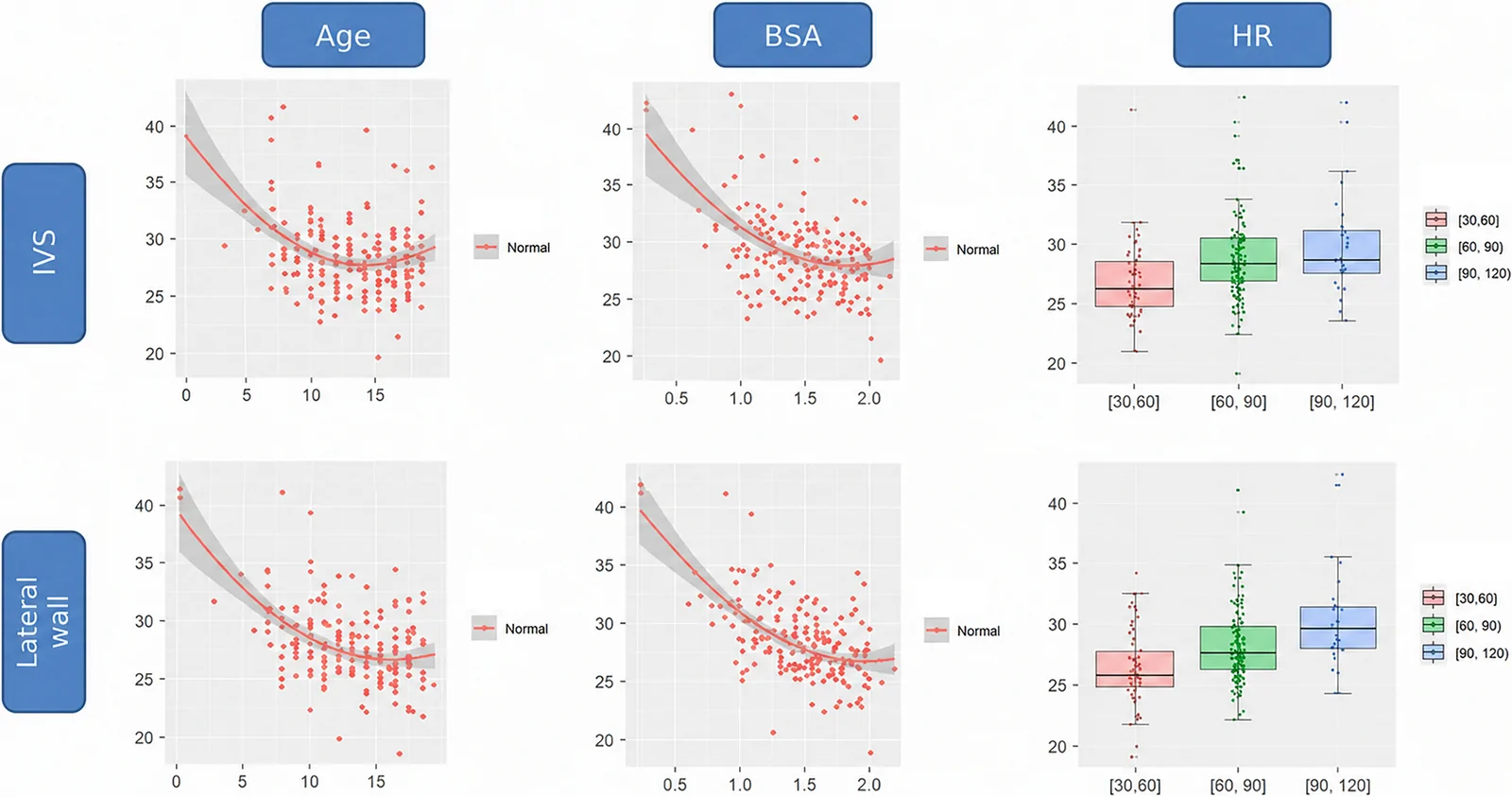

**Fig. 4**

**Figure:**
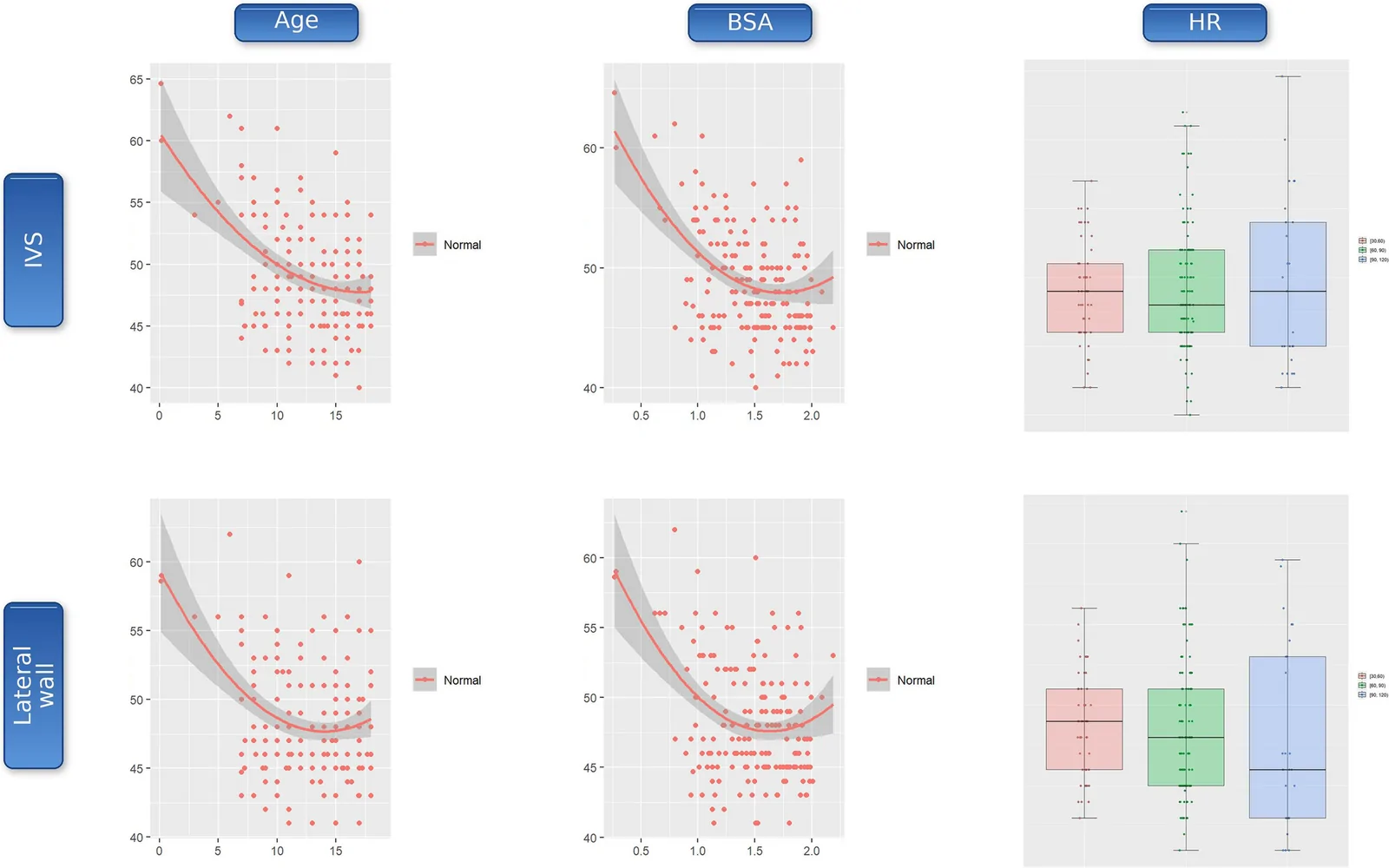

**Fig. 5**

The original article has been corrected.

