# Supplementary material for: Impact of age, heart rate, and body surface area on myocardial native T1, T2, and extracellular volume fraction in pediatric patients at 1.5 Tesla cardiac magnetic resonance imaging
Source: Pediatr Radiol. 2026 Jul 30;56(12):2837–48. doi: 10.1007/s00247-026-06714-y (PMC13759569; doi:10.1007/s00247-026-06714-y)
Supplement: Supplementary file 1 [file 247_2026_6714_MOESM1_ESM.docx]

|  | **Age** | | **BSA** | |
| --- | --- | --- | --- | --- |
| **Native T1** | IVS | Free wall | IVS | Free wall |
| Quadratic equation | Y = 1,141.7 – 20.4X + 0.75 X^2^ | Y = 1,145 – 20.4 X + 0.6 X^2^ | Y = 1,169.6 – 184.9 X + 50.3 X^2^ | Y = 1,193 – 220.6 X + 56.1 X^2^ |
| Adjusted R^2^ | 0.168 | 0.290 | 0.205 | 0.368 |
| p value | 0.001 | 0.001 | 0.001 | 0.001 |
| **ECV** |  |  |  |  |
| Quadratic equation | Y = 39.4 - 1.8 X + 0.06 X^2^ | Y = 38.7 – 1.6 X + 0.04 X^2^ | Y = 41.5 – 15.6 X + 3.67 X^2^ | Y = 42.2 – 17.3 X + 3.85 X^2^ |
| Adjusted R^2^ | 0.260 | 0.382 | 0.328 | 0.436 |
| p value | 0.001 | 0.001 | 0.001 | 0.001 |
| **T2** |  |  |  |  |
| Quadratic equation | Y = 60.65 -1.5 X + 0.04 X^2^ | Y = 59.4 – 1.7 X + 0.06 X^2^ | Y = 66.8 – 21.8 X + 6.30 X^2^ | Y = 64 – 20.2 X + 6.19 X^2^ |
| Adjusted R^2^ | 0.163 | 0.116 | 0.168 | 0.127 |
| p value | 0.001 | 0.001 | 0.001 | 0.001 |
